# An audit of primary surgical treatment for women with ovarian cancer referred to a cancer centre

**DOI:** 10.1038/sj.bjc.6690376

**Published:** 1999-05-01

**Authors:** P S Sengupta, G C Jayson, R J Slade, A Eardley, J A Radford

**Affiliations:** Cancer Research Campaign and University of Manchester Department of Medical Oncology, Christie Hospital National Health Trust, Wilmslow Road Withington, Manchester M20 4BX, UK; Obstetrics & Gynaecology Directorate, Hope Hospital, Salford Royal Hospitals National Health Trust, Stott Lane, Salford, M6 8HD, UK; Clinical Audit & Quality Assurance Facilitator, Christie Hospital National Health Trust, Withington, Manchester M20 4BX, UK

**Keywords:** audit, ovarian cancer, surgery, staging

## Abstract

Ovarian cancer is the commonest cause of gynaecological cancer death in the UK, and guidelines for initial surgery and staging of this disease are widely available. We report a retrospective audit of the surgical management of patients with newly diagnosed ovarian cancer referred to the Christie Cancer Centre in Manchester in 1996. The aim was to assess compliance with surgical guidelines. The authors found that the majority of patients (92%) presented via an outpatient clinic and for these individuals surgery was therefore elective. This mode of presentation should allow management by a small number of dedicated gynaecologists at each hospital, but up to seven consultants in each hospital performed surgery on a relatively small number of patients. Furthermore, less than half the patients underwent the recommended surgical procedure. Although some patients may have ‘inoperable’ disease, these data suggest that a greater compliance with national and international guidelines are required to provide an optimal level of care. © 1999 Cancer Research Campaign


British Journal of Cancer (1999) 80(3/4), 444–447
© 1999 Cancer Research Campaign
Article no. bjoc.1998.0376

### An audit of primary surgical treatment for women with ovarian cancer referred to a cancer centre

**PS Sengupta[1], GC Jayson[1], RJ Slade[2], A Eardley[3] and JA Radford[1]**

[1]Cancer Research Campaign and University of Manchester Department of Medical Oncology, Christie Hospital National Health Trust, Wilmslow Road, Withington, Manchester M20 4BX, UK; [2]Obstetrics & Gynaecology Directorate, Hope Hospital, Salford Royal Hospitals National Health Trust, Stott Lane, Salford M6 8HD, UK; [3]Clinical Audit & Quality Assurance Facilitator, Christie Hospital National Health Trust, Withington, Manchester M20 4BX, UK

**Summary** Ovarian cancer is the commonest cause of gynaecological cancer death in the UK, and guidelines for initial surgery and staging of this disease are widely available. We report a retrospective audit of the surgical management of patients with newly diagnosed ovarian cancer referred to the Christie Cancer Centre in Manchester in 1996. The aim was to assess compliance with surgical guidelines. The authors found that the majority of patients (92%) presented via an outpatient clinic and for these individuals surgery was therefore elective. This mode of presentation should allow management by a small number of dedicated gynaecologists at each hospital, but up to seven consultants in each hospital performed surgery on a relatively small number of patients. Furthermore, less than half the patients underwent the recommended surgical procedure. Although some patients may have ‘inoperable’ disease, these data suggest that a greater compliance with national and international guidelines are required to provide an optimal level of care.

**Keywords:** audit; ovarian cancer; surgery; staging

Ovarian cancer is the commonest cause of gynaecological cancer-related death in the UK (Association of Cancer Physicians, 1994). In the northwest of England, the annual incidence is 183 cases per million and, between 1989 and 1991, 1085 cases were recorded in the North West Cancer Registry (unpublished data).

The optimal management of ovarian carcinoma is defined in the Scott Report (Scott, 1991) and 1993 consensus statements on advanced epithelial ovarian cancer (Allen et al, 1993). These state that an experienced gynaecological surgeon should attempt optimum cytoreduction (debulking), including total abdominal hysterectomy (TAH), bilateral salpingo-oophorectomy (BSO) and infracolic omentectomy. Those with suspected early FIGO (International Federation of Obstetricians & Gynaecologists) stage disease (Shepherd, 1992) should have ascitic fluid or peritoneal washings sent for cytological evaluation, together with random peritoneal biopsies and diaphragmatic scrapings. Adhesions or suspicious areas should be biopsied, and the liver and diaphragm palpated to assess the extent of the disease. Some have also advocated routine retroperitoneal lymphadenectomy. Those with more advanced disease should undergo debulking of all macroscopic disease. Patients should then have further appropriate staging investigations and should receive chemotherapy from a non-surgical oncologist who is trained in the field (Scott, 1991; Allen et al, 1993). A multidisciplinary approach has been associated with improved survival (Junor et al, 1994).

We have performed a retrospective audit of the initial surgical management of patients with newly diagnosed epithelial ovarian carcinoma referred for chemotherapy to the Department of Medical Oncology at the Christie Cancer Centre in 1996. The aim was to assess the compliance with surgical guidelines for the management of epithelial ovarian carcinoma.

*Received 23 July 1998*
*Revised 18 November 1998*
*Accepted 4 December 1998*

*Correspondence to:* PS Sengupta

### METHODS

Currently, all primary surgery for ovarian cancer in northwest England is performed in district general hospitals. Women with ovarian cancer can then be referred to a non-surgical oncologist at the Christie Cancer Centre. All cases of ovarian cancer referred to the Department of Medical Oncology during 1996 were included in the audit, which was conducted using standard criteria (National Centre for Clinical Audit, 1997).

A retrospective review of the Christie Hospital case notes, and referring hospital correspondence, pathology reports and operation notes, was performed to determine:

1. The presentation and pre-operative investigation of patients.
2. The surgery performed including a stage-specific (FIGO stage I vs stages II–IV) analysis of surgery.
3. The information recorded in the operation notes.

### RESULTS

In 1996, the Department of Medical Oncology received 87 referrals for the post-operative management of ovarian cancer and the case notes of 85 of these patients were available. Table 1 records the characteristics of these patients.

### Clinical presentation and pre-operative investigations

The mode of presentation of ovarian cancer was largely sub-acute. Seventy-eight of the 85 (92%) patients presented with symptoms that were first assessed in the out-patient clinic and then proceeded to elective surgical management. Seven cases (8%) presented acutely, although four of these underwent detailed pre-operative radiological assessment before elective surgery.

Abdomino-pelvic ultrasound scan (USS) or computerized tomography (CT) scan were performed in 60 cases (15 women had both; total 74%) and two women underwent magnetic resonance imaging. Reference to the pre- or post-operative serum concentration of the tumour marker CA125 was made in only 14 cases before referral to the Christie Cancer Centre.

### Operating surgeons

Eighty-three patients were operated on by 31 gynaecologists and four general surgeons from 14 district general hospitals (range 1–17 patients per hospital, median eight). Two patients were excluded since one had refused surgery and another had recurrent disease.

The number of consultants in each hospital who operated on a patient with ovarian cancer was between one and seven (median three). The range of patients operated on by each consultant was 1–11 (median one). Hence, in one hospital a single consultant operated on 11 cases of ovarian cancer while in another seven consultants operated on 17 patients (Figure 1).

### Surgery

The recommendations for surgery in cases of suspected ovarian cancer include a vertical abdominal incision, total abdominal hysterectomy, bilateral salpingo-oophorectomy and omentectomy.

Clearly recorded details of the initial incision were found for 75 patients. Five patients had a transverse suprapubic (pfannenstiel) incision, of whom four had undergone detailed pre-operative radiological investigations. Otherwise the recommended vertical abdominal incision was made in 93% of this group.

Further surgical details were available for the 83 patients, some of whom had undergone previous gynaecological surgery. TAH was performed in 48 of 76 patients who had not previously undergone hysterectomy (63%). BSO (or oophorectomy if an ovary had been previously removed) was performed in 61 out of 81 patients (75%). Infracolic omentectomy was performed in 41 of 83 cases (49%). Overall, 76 patients had not undergone previous gynaecological surgery, and 32 (42%) of these underwent the full recommended surgical procedure. Post-operative CT scanning showed no evidence of residual disease in 13 of these 32 patients.

**Table 1** Characteristics of patients referred with ovarian carcinoma ($n$ = 85)

| Characteristic | No. of patients | % |
|---|---|---|
| Age (median = 59 years) | | |
| <35 | 4 | 5 |
| 36–45 | 10 | 11 |
| >45 | 71 | 84 |
| FIGO stage | | |
| I | 24 | 28 |
| II | 5 | 6 |
| III | 38 | 45 |
| IV | 18 | 21 |
| Bulk residual disease (>1.5 cm) | ($n$ = 84) | (% of each stage) |
| I | 0/24 | 0 |
| II | 4 / 5 | 80 |
| III | 28 / 37 | 76 |
| IV | 15 / 18 | 83 |
| Histologic type | | |
| Benign (on pathology review) | 2 | 2 |
| Not otherwise specified | 13 | 15 |
| Clear cell | 5 | 6 |
| Endometrioid | 16 | 19 |
| Mucinous | 10 | 12 |
| Serous | 20 | 24 |
| Other | 11 | 13 |
| Borderline malignancy/pseudomyxoma | 8 | 9 |
| Tumour differentiation (excluding borderline cases) | ($n$ = 77) | (% of 77) |
| Well | 13 | 17 |
| Moderately | 19 | 25 |
| Poor/undifferentiated | 30 | 39 |
| Not known | 13 | 17 |
| Benign | 2 | 3 |

### Stage-specific surgery

Twenty-four women had FIGO stage I ovarian cancer. The abdominal incision was determined in 21 patients and a vertical incision confirmed in 18 (86%). Ten of 22 patients (two had previous surgery) underwent TAH, BSO and omentectomy (45%). Eleven patients (46%) had peritoneal fluid or washings taken for cytological examination. Only two patients (8%) had peritoneal biopsies performed for detection of occult disease (both of suspicious areas). There were no biopsies of adhesions, diaphragmatic scrapings, or lymph node sampling.

Sixty-one patients had at least stage II ovarian cancer. Of these, 22 patients (36%) underwent TAH, BSO and omentectomy (or the equivalent when previous gynaecological surgery had been performed). At post-operative evaluation by CT scan, five patients (8%) had no residual disease while 48 (79%) had bulk residual disease (defined as more than 1.5 cm diameter).

Both requirement for chemotherapy and the prognosis were determined by the FIGO stage and extent of residual disease. This was determined by integrating information including histology, radiology and the recorded surgical findings. Of 83 patients who had surgery, the FIGO stage was recorded in 20 cases, and a statement concerning residual disease made in 45 cases.

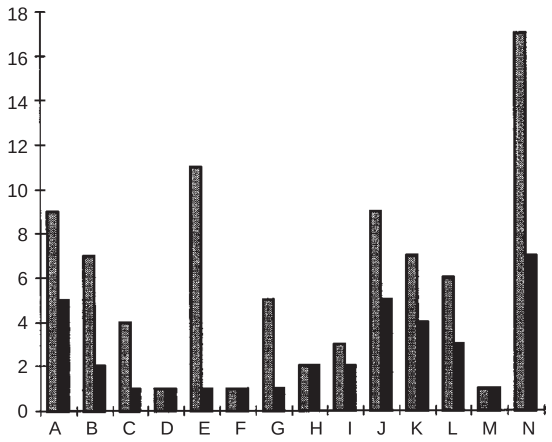

**Figure 1** Number of patients (grey bars) and surgeons (black bars) in each referring hospital (letters)

### DISCUSSION

The initial management of patients with ovarian cancer is surgical. This aims to establish the histological diagnosis, achieve optimal cytoreduction and determine the stage of the disease. Guidelines (Scott, 1991; Allen et al 1993) recommend that patients with suspected ovarian cancer should undergo total abdominal hysterectomy, bilateral salpingo-oophorectomy, infracolic omentectomy and removal of all other visible tumour through a vertical abdominal incision. For those patients who are believed to have early stage disease, peritoneal fluid or washings should be sent for cytological evaluation. Random biopsies of the peritoneum, diaphragmatic scrapings, and palpation of viscera including the liver should also be performed. Although sometimes recommended, lymph node sampling in stage I disease is not a standard procedure in the UK at present.

As a tertiary referral centre, the Christie Cancer Centre serves the northwest region of England. The surgical workload comprises patients with relapsed disease or surgery after chemotherapy. Currently, all primary surgery in the region is performed in district general hospitals. A minority of women with ovarian cancer are then referred to a non-surgical oncologist. Between 1989 and 1991, the Christie Cancer Centre was referred 436 patients with ovarian cancer, representing 40% of registered cases (unpublished data).

### Patient selection

This audit reports the surgical management of all the patients with ovarian cancer referred to the Medical Oncology Department of this regional Cancer Centre in 1996, a cohort corresponding to approximately a quarter of the total number of regional cases. Although the numbers are quite small, it is likely that this group is representative of those who are receiving optimal treatment, as the guidelines state that referral to a non-surgical oncologist should always be considered following the diagnosis of ovarian cancer. A number of important findings were made.

### Clinical presentation and investigation

Most women presented as out-patients and were assessed radiologically before an elective laparotomy. Seven cases presented acutely, but four were still investigated with detailed radiological assessment before surgery was considered. The acute presentation of ovarian carcinoma is therefore unusual and so the guideline recommendations, which state that an experienced gynaecological surgeon should perform the operation, do not appear unreasonable. A further consequence of this mode of presentation is that a small group of nominated consultants could specialize in ovarian cancer. However, the data show that up to seven consultants in each hospital operated on patients with ovarian cancer.

### Surgery

Current recommendations suggest that patients should undergo TAH, BSO and omentectomy, yet only 32 of 76 patients who had not undergone previous gynaecological surgery underwent this procedure. Surgery to this extent may not be possible because of difficulty with the dissection of normal structures from tumour. In stage I disease, however, where disease is confined to the ovaries, technical difficulties are likely to be minimal but despite this we found that only ten of 22 patients with stage I disease underwent the full recommended procedures. Furthermore, in cases where a hysterectomy is performed, an omentectomy should be possible, but we recorded a TAH and BSO in more than 60% of cases and an omentectomy in less than 50%. Conservative surgery might have occurred because operators did not consider a diagnosis of ovarian cancer, but 74% of patients underwent detailed pre-operative radiological evaluation which might have been expected to suggest pelvic malignancy. In addition, most of the patients had a vertical abdominal incision, which is not routine in gynaecological surgery, implying that a sinister pathology was, in fact, suspected.

### Staging procedures

The recommended staging procedures for suspected stage I ovarian carcinoma include microscopy of ascites or peritoneal washings for malignant cells, with random peritoneal biopsies to exclude occult disease. However, the data show that less than half the cases underwent these procedures and only two patients had peritoneal biopsies performed. Conservative surgery, where the uterus and normal ovary are preserved, can be practised in younger patients who wish to preserve their fertility. Nevertheless, only four patients in this series were less than 35 years old (Table 1).

### Recording of operative findings

Statements concerning the FIGO stage and the presence of residual disease in referral letters or in the operation notes contribute to advice given concerning prognosis and the decision concerning further medical management. In this group of patients, only 44 of 83 referral letters or operation notes contained information about residual disease, and only a quarter stated the FIGO stage.

### CONCLUSIONS

This audit has shown that in northwest England many consultants are operating on a few patients with ovarian cancer. This is not because patients present as emergencies since most are seen as out-patients and evaluated with detailed radiological assessments before operation. In addition, many patients are not undergoing the recommended surgery or staging procedures, although in part this may be due to patients having genuinely inoperable disease. The Royal College of Obstetricians and Gynaecologists, and British Gynaecological Cancer Society (1996) have advocated the development of specialist gynaecological oncology centres and units. These developments are intended to improve compliance with management guidelines, and will be the subject of a further audit following implementation.

The results of this study were presented both as an oral presentation and poster at the Bristol Meeting of the British Gynaecological Cancer Society, 7–8 November 1997, and as an oral presentation at the Manchester Ovarian Group Meeting, 15 January 1998.

### REFERENCES

Association of Cancer Physicians (1994) Review of the pattern of cancer services in England and Wales. Association of Cancer Physicians, Surrey, United Kingdom

Allen DG, Baak J, Belpomme D, Berek JS, Bertelsen K, ten Bokkel Huinink WW, van der Burg ME, Calvert AH, Conte PF, Dauplat J, Eisenhauer EA, Favalli G, Hacker NF, Hamilton TC, Hansen HH, Hansen M, van Houwelingen HC, Kaye

© Cancer Research Campaign 1999

SB, Levin L, Lund B, Neijt JP, Ozols RF, Piccart MJ, Rustin GJS, Sessa C, Soutter WP, Thigpen JT, Tropé C, Vermorken JB and de Vries EGE (1993). Advanced epithelial ovarian cancer: 1993 consensus statements. *Ann Oncol* **4**: S83–S88

Royal College of Obstetricians and Gynaecologists and British Gynaecological Cancer Society (1996) A joint working group response to ‘A Policy Framework for Commissioning Cancer Services’. Royal College of Obstericians and Gynaecologists and British Gynaecological Cancer Society, London, UK

Junor EJ, Hole DJ and Gillis CR (1994) Management of ovarian cancer: referral to a multidisciplinary team matters. *Br J Cancer* **70**: 363–370

National Centre for Clinical Audit (1997) Information for better healthcare. Key points from audit literature related to criteria for clinical audit. Clinical audit action pack, version 2. National Centre for Clinical Audit Publications, London, UK

Scott JS (1991) Management of ovarian cancer. Current clinical practices. Report of a working group. Standing Subcommittee on Cancer of the Standing Medical Advisory Committee, London, UK

Shepherd JH (1992) Revised FIGO staging for gynaecological cancer. *Br J Obstet Gynaecol* **96**: 889–892

© Cancer Research Campaign 1999
